# Left ventricular pacing induced polymorphic ventricular tachycardia via the adaptive left ventricle pacing algorithm

**DOI:** 10.1002/ccr3.2940

**Published:** 2020-05-26

**Authors:** Fouad Khalil, Freddy Del‐Carpio Munoz, Abhishek Deshmukh, Ammar M. Killu

**Affiliations:** ^1^ Mayo Clinic Rochester Minnesota USA

**Keywords:** cardiac resynchronization therapy, his‐bundle pacing, LV pacing, polymorphic ventricular tachycardia, proarrhythmia, QT prolongation, transmural dispersion of repolarization

## Abstract

Providers should be aware of the possibility of cardiac resynchronization therapy‐related proarrhythmia which could be life‐threatening. His‐bundle pacing may serve as an alternative, more physiological, option in the management as it preserves the normal sequence of depolarization from the septum to the lateral wall, and from endocardium to epicardium.

## Graphical abstract

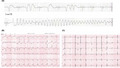

## 1 INTRODUCTION

Implantable cardioverter defibrillator (ICD) therapy is associated with a reduction in sudden arrhythmic death.^1^ Cardiac resynchronization therapy (CRT) with (CRT‐D) or without (CRT‐P) defibrillator is also associated with improvements in survival, heart failure symptoms, and quality of life in those with left bundle branch block and prolonged QRS duration.^2^ Though rare, CRT‐induced proarrhythmia represents a potentially life‐threatening complication of CRT.^3^ Herein, we describe a report of adaptive LV algorithm induced polymorphic ventricular tachycardia (VT) immediately after the initiation of CRT‐P despite excellent ECG result. The patient required multiple antiarrhythmic agents, turning off LV pacing and ultimately His‐bundle pacing with an ICD.

## 2 CASE PRESENTATION

**FIGURE 1:**
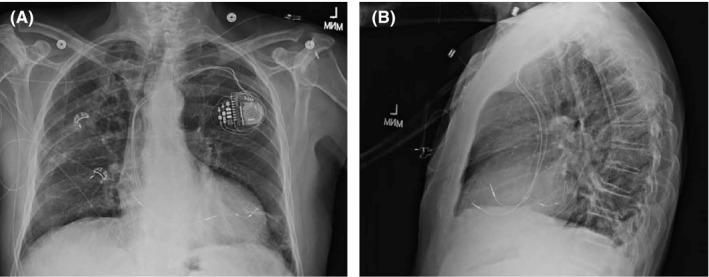
Post‐CRT‐P chest X‐ray, anteroposterior (A) and lateral (B) views show left‐sided CRT device with leads in the right atrial appendage, right ventricle, and a lateral cardiac vein

An 84‐year‐old man with systolic heart failure, severely depressed left ventricular function (EF 20%), functional class III, and frequent ventricular ectopy treated with amiodarone was referred for CRT‐P (declined ICD). He had a history of idiopathic dilated cardiomyopathy, diabetes, hypertension, sleep apnea, and hypothyroidism. Baseline ECG demonstrated normal sinus rhythm with LBBB; QRS duration was 150 ms, and corrected QT interval (QTc) was 450 ms when accounting for LBBB in which the prolongation of the QRS due to the bundle branch block was subtracted from the (QTc) and QT dispersion (QTd) was 48 ms.^4^ The procedure was acutely uncomplicated, and the LV lead was placed at a suitable posterolateral branch of the coronary sinus with excellent pacing thresholds. He underwent placement of a Medtronic Percepta CRT‐P (Medtronic Capsure Fix 4076 RA and RV leads and Medtronic Attain Performa 4598). RA pacing threshold was 0.8 V at 0.5 ms, the RV pacing threshold was 0.3 V at 0.5 ms, and the LV lead pacing threshold was 01.5 V at 0.5 ms (Figure 1).

**FIGURE 2:**
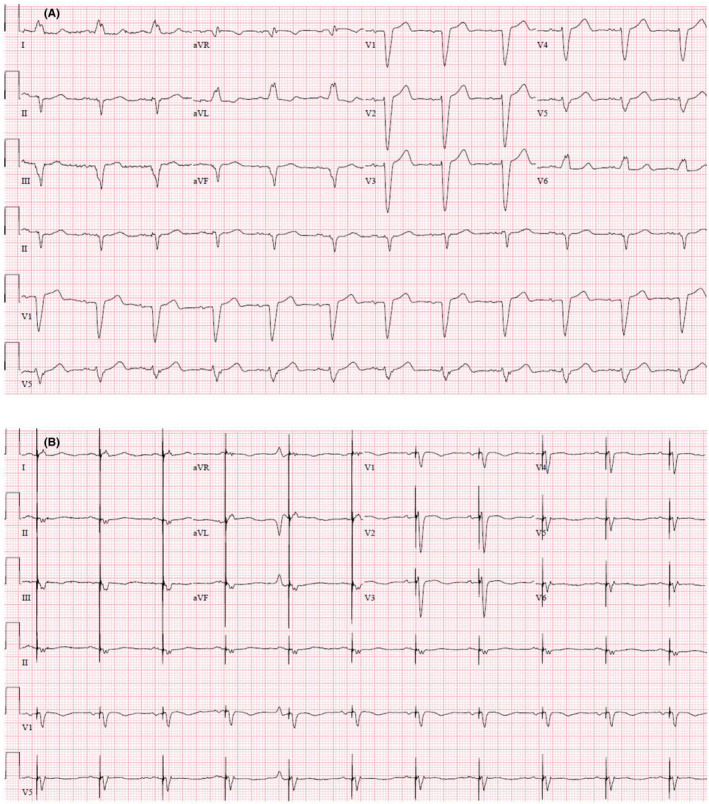
Pre‐ and post‐CRT‐P ECG. A, Baseline ECG shows normal sinus rhythm with left bundle branch block, QRS (150 ms), Prolonged QT (QTc 450 ms) and (QTd 48 ms). B, Post‐CRT‐P ECG shows ventricular pacing, QRS (140 ms), QTc (465 ms) and QTd (96 ms)

Postprocedure ECG showed a shorter QRS duration (140 ms), the QTc was 465 ms with ventricular pacing, and QTd was 96 ms (Figure 2).

The patient was discharged on optimal medical therapy without any QT‐prolonging medications except amiodarone.

**FIGURE 3:**
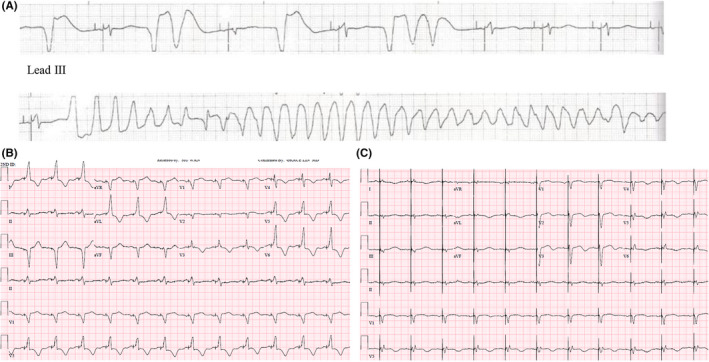
A, ECG (Lead III) shows the initiation of polymorphic ventricular tachycardia (PMVT) by premature ventricular contraction (PVC) with “R on T” in the setting of prolonged QT interval. B, Twelve lead surface ECG shows a markedly prolonged corrected QT interval of 655 ms in the setting of adaptive LV pacing and QTd (23 ms). C, Post‐HBP ECG shows dual chamber pace maker with corrected QT <500 ms when accounting for the paced QRS duration and QTd (4 ms)

The day of dismissal, he had a syncopal event while at home. Emergency medical services were contacted, and due to recurrent syncope, he was admitted to the coronary care unit (CCU). Upon admission to the cardiac care unit, he had repetitive episodes of sustained polymorphic ventricular tachycardia (PMVT). His ECG showed considerable QT prolongation from baseline (QTc 655 ms and QTd 23 ms). (Figure 3) He was started on intravenous lidocaine. Device interrogation was performed and identified 25 ventricular high rate episodes, all of which had a similar initiation, in which PVC following a paced beat induced PMVT. (Figure 3) Due to concern for pacing proarrhythmia, LV pacing was turned off and the device set to atrial pacing at a rate of 90 beats/minute which was successful in preventing PMVT recurrence.

Patient medication list did not include any QT‐prolonging medication asides from amiodarone. While this can increase QT interval, the risk of proarrhythmia is exceptionally low.^5^, ^6^, ^7^ Furthermore, the QT interval prior to CRT‐P was stable and drastically increased following CRT‐P despite no change in amiodarone dosing. Serum electrolytes were within normal limits (K 3.7 [ref: 3.6‐5.2 mmol/L], Ca 9 [ref: 8.8‐10.2 mg/dL], and Mg 2.2 [ref: 1.7‐2.3 mg/dL]).

**FIGURE 4:**
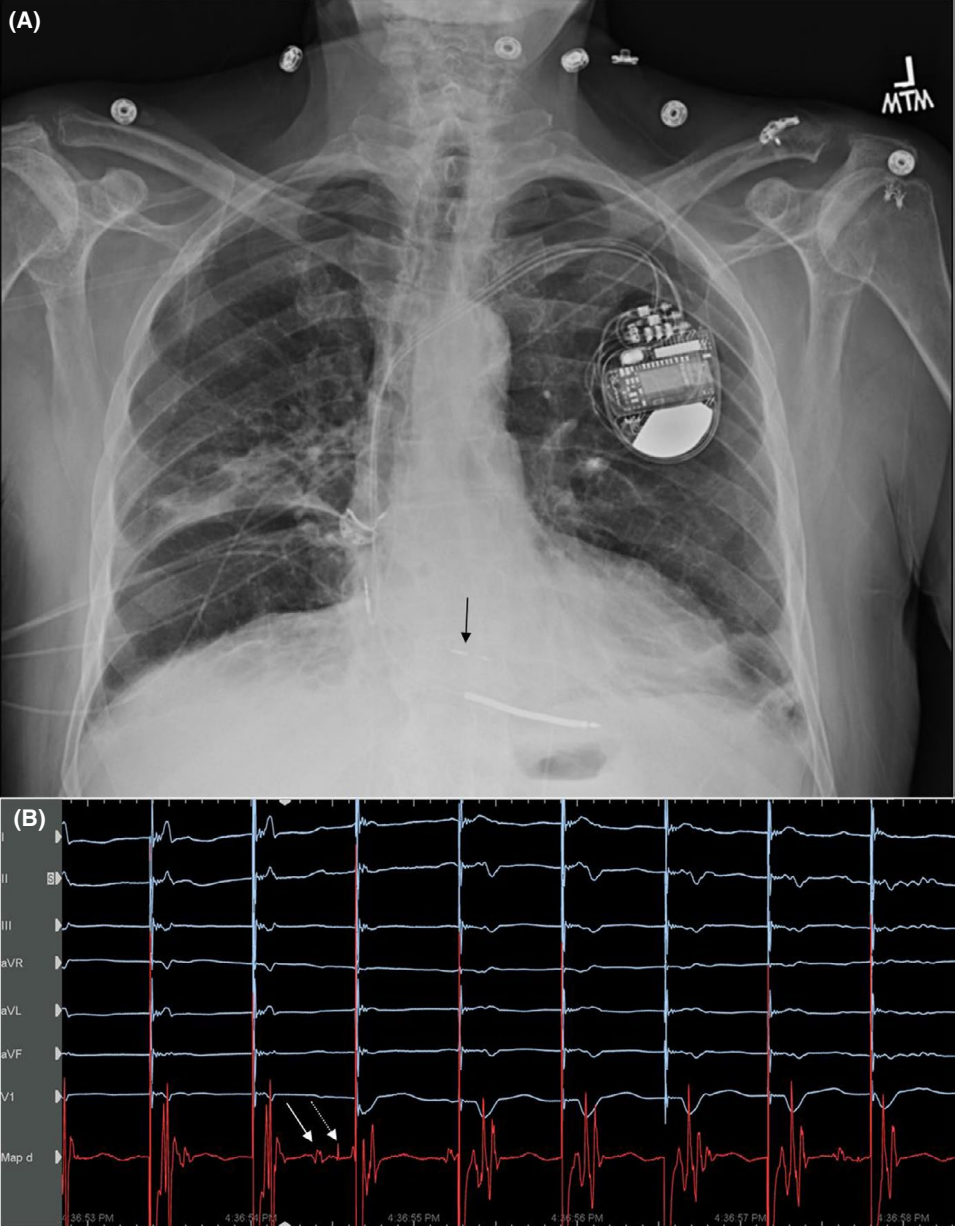
A, PA chest radiograph demonstrating pacing lead in the His‐bundle region (black arrow). B, Intracardiac lead recording demonstrating His‐bundle pacing at location depicted in panel A. The first four beats have direct His capture with a short isoelectric baseline and narrow QRS. The 5th beat demonstrates fusion. An atrial electrogram (solid arrow) followed by His‐bundle electrogram (dashed arrow) is seen. The QRS has a fused morphology between His‐bundle capture and loss of His‐bundle capture. With continued pacing, loss of His‐bundle capture with myocardial capture alone is noted. This was a repeated occurrence

A transthoracic echocardiogram showed no regional wall motion abnormalities. An emergent coronary angiogram was performed and demonstrated no significant coronary artery disease. Due to concern for recurrent PMVT with LV pacing, it was decided to revise his system and attempt His‐bundle pacing. The patient now also agreed to undergo ICD implantation. He underwent placement of a Medtronic Claria MRI CRT‐D (Medtronic Capsure Fix Novus 4076 RA lead, Medtronic 6935M ICD Sprint Quattro ICD lead and Medtronic 3830 SelectSecure LV lead). The CS and RV pacing leads were removed with placement of an RV shock lead and pacing lead in the His‐bundle position (inserted into the LV port of the CRT‐D device). RA pacing threshold was 0.75 V at 0.4 ms, the RV pacing threshold was 0.5 V at 0.4 ms, and the His lead pacing threshold was 0.5 V at 1.0 ms Though we could obtain selective His capture for brief periods, ultimately, only nonselective capture was possible, achieving a final QRS duration of 145 ms (Figure 4).

On routine 3‐month postimplant clinic visit, the patient had satisfactory device function with ventricular pacing occurring 99% of the time. There were no further episodes of ventricular arrhythmias. LVEF had increased from 20% to 32% with concomitant decrease in NT‐proBNP (from 3929 to 1201 pg/mL, reference range ≤138 pg/mL). ECG showed corrected QT <500 ms when accounting for the paced QRS duration and QTd was 4 ms. (Figure 3).

## 3 DISCUSSION

Following the first reported success of CRT in 1995,^8^ biventricular pacing (BiVP) has become an important therapy in the management of congestive heart failure. CRT can significantly improve cardiac function, NYHA functional class, exercise tolerance, quality of life, decrease heart failure hospitalizations and improve survival in patients with heart failure, systolic dysfunction, and left‐sided conduction delay. By normalizing delayed intraventricular conduction, CRT reduces LV septal to lateral wall dyssynchrony, decreases myocardial oxygen consumption, enhances LV diastolic filling, and increases stroke volume.^9^ Although a great deal of attention has been focused on exhibiting its clinical benefits, little attention has been focused on the consequences of the abnormal sequence of ventricular activation. A few case reports have described CRT‐induced VT, ventricular fibrillation, and Torsade de Pointes.^10^, ^11^ Although the underlying pathophysiology for proarrhythmia has not been clearly established, the most likely mechanism is the repolarization change induced by BiVP. Ventricular myocardium normally depolarizes from endocardium to epicardium, but repolarization occurs in the reverse direction. Reversal of the normal activation sequence can prolong the QT interval by enhancing the existing transmural dispersion of repolarization (TDR). With BiVP, epicardial action potential depolarizes and repolarizes earlier and the subendocardial M cells with the longest duration of action potential depolarize and repolarize later compared with the normal endocardium to epicardium sequence of activation, creating an adequate substrate for reentrant arrhythmias.^12^ Also, it is postulated that pacing from a left ventricular (LV) lead positioned on an epicardial scar can facilitate re‐entrant VT.^13^ Therefore, imaging has a very important role in planning of CRT therapy. Cardiac magnetic resonance (CMR) may be used to help guide lead placement due to its ability to define coronary vein anatomy and delineation of myocardial scar.^14^ Furthermore, studies have suggested that myocardial scar extent could be predictive of CRT response.^15^ This is of special importance in nonischemic cardiomyopathy where CRT‐induced proarrhythmia is more likely and substrates tend to be more complex.^16^

Management of device‐related proarrhythmia can be challenging—while management options include antiarrhythmic drug therapy and ablation therapy, oftentimes, CRT reprogramming or inactivation needs to performed.

As our case demonstrates, the patient was initiated on IV lidocaine and the LV pacing was turned off with a reduction in QT interval. After excluding the option of surgical placement of LV lead which could also increase the risk of proarrhythmia, we had the following options:

- Continue with medications and abandon efforts at resynchronization therapy. Given his marked limitations and impaired quality of life, this choice was excluded.
- Endocardial placing of an LV lead using the trans‐septal approach. With this option, the patient would need lifelong anticoagulation with potential risks of thromboembolism and stroke.^17^
- Leadless left ventricular endocardial pacing using the WiSE‐CRT system. The WiSE‐CRT system is still under study. The patient was not interested in this option.
- His‐bundle pacing (HBP), which compared to the other ventricular pacing modalities, tends to preserve most the normal ventricular activation sequence. This was identified as the most suitable alternative.

The patient was deemed candidate for a secondary prevention ICD (and was a primary prevention candidate previously, but refused). As such, he underwent ICD implantation in addition to HBP and no further VT was reported.

His‐bundle pacing utilizes the native His‐Purkinje conduction system, preserving the normal sequence of ventricular depolarization from the septum to lateral wall and endocardium to epicardium, which is a physiological sequence of electrical activation; it thus maintains electrical and mechanical synchrony without reversing the direction of activation as in BVP. Also, HBP was found to reduce T peak‐T end (Tp‐Te) duration, a measure of repolarization dispersion that constitutes a marker for arrhythmia risk.^18^ Selective HBP refers to the capture of His ‐Bundle only with resulting QRS identical to patient's native QRS due to conduction through His‐Purkinje system only. On the other hand, nonselective HBP refers to the additional capture of septal myocardium with resulting initial slurring and widening of QRS due to RV myocardial pre‐excitation. Studies have demonstrated that both techniques can restore normal electrical and mechanical outcomes with similar outcomes regarding heart failure hospitalization and death.^19^, ^20^

Many recent studies have demonstrated the potential of HBP in patients with underlying cardiomyopathy and bundle branch block.^21^, ^22^ However, little attention has been focused on HBP as an option for pacing‐related proarrhythmias. Sofi et al have reported a case that highlights the potential of HBP as an option for management proarrythmia.^23^

In line with other reports, our case raises a serious and concerning issue of CRT causing acute proarrhythmia. It would be prudent for providers initiating CRT to be aware of the rare possibility of proarrhythmic effects.

While our case describes the unpredictable and life‐threatening proarrhythmia associated with LV pacing, it also demonstrates the potential of HBP as an alternative for patients with pacing associated proarrhythmia.

## CONFLICT OF INTEREST

The authors declare no conflict of interest.

## AUTHOR CONTRIBUTIONS

FK: involved in conception and design. FK and AK: drafted the article. FK, AK, FD‐M, and AD: critically revised and approved the article.
